# Evaporation Rate of Water as a Function of a Magnetic Field and Field Gradient

**DOI:** 10.3390/ijms131216916

**Published:** 2012-12-11

**Authors:** Yun-Zhu Guo, Da-Chuan Yin, Hui-Ling Cao, Jian-Yu Shi, Chen-Yan Zhang, Yong-Ming Liu, Huan-Huan Huang, Yue Liu, Yan Wang, Wei-Hong Guo, Ai-Rong Qian, Peng Shang

**Affiliations:** Key Laboratory for Space Bioscience & Biotechnology, School of Life Sciences, Northwestern Polytechnical University, Xi’an 710072, Shaanxi, China; E-Mails: guoyunzhu@mail.nwpu.edu.cn (Y.-Z.G.); hlcao@mail.nwpu.edu.cn (H.-L.C.); jianyushi@nwpu.edu.cn (J.-Y.S.); zhcy925@gmail.com (C.-Y.Z.); auliuym@163.com (Y.-M.L.); h87928@gmail.com (H.-H.H.); chou820@foxmail.com (Y.L.); wangyan1987020745@163.com (Y.W.); gwh910@yahoo.com.cn (W.-H.G.); qianair@nwpu.edu.cn (A.-R.Q.); shangpeng@nwpu.edu.cn (P.S.)

**Keywords:** molecular water, evaporation, magnetic field, field gradient, protein crystallization, hydrogen bond, van der Waals force

## Abstract

The effect of magnetic fields on water is still a highly controversial topic despite the vast amount of research devoted to this topic in past decades. Enhanced water evaporation in a magnetic field, however, is less disputed. The underlying mechanism for this phenomenon has been investigated in previous studies. In this paper, we present an investigation of the evaporation of water in a large gradient magnetic field. The evaporation of pure water at simulated gravity positions (0 gravity level (ab. g), 1 g, 1.56 g and 1.96 g) in a superconducting magnet was compared with that in the absence of the magnetic field. The results showed that the evaporation of water was indeed faster in the magnetic field than in the absence of the magnetic field. Furthermore, the amount of water evaporation differed depending on the position of the sample within the magnetic field. In particular, the evaporation at 0 g was clearly faster than that at other positions. The results are discussed from the point of view of the evaporation surface area of the water/air interface and the convection induced by the magnetization force due to the difference in the magnetic susceptibility of water vapor and the surrounding air.

## 1. Introduction

Since the middle of the last century, many studies have investigated the effect of a magnetic field on water, but the results of these studies were often contradictory. The issue was raised in the early 1950s when commercial “water conditioners” using permanent magnets were sold as a unit [1]. It was said that such water conditioners could remove old scale and prevent new scale from forming in water pipes or water boilers if the water passed through a magnetic field created by the permanent magnet. However, a report in 1958 showed that water conditioners, or even a much stronger magnetic field, could not alter the scale-forming properties of water [1]. Since that time, there have been many contradictory arguments opinions on the effect of a magnetic field on water. For example, in 1985, Klaus J. Kronenberg showed that the crystals grown from the mineral content in water could be changed from dendritic crystals into the separate disc-shaped crystals with treatment of the water by magnetic fields [2]. However, other researchers found the phenomenon in that report to be suspect [3,4].

Most of the arguments are actually related to the chemical substances in the water and not related to the properties of water itself. Other studies that have focused on the effect of a magnetic field on the physical and chemical properties of pure water have yielded inconsistent research results. For example, Cai *et al*. [5] pointed out that a magnetic field can decrease the surface tension while increasing the viscosity of water, whereas Toledo *et al.*[6] concluded that a magnetic field increases the surface tension.

These discrepancies, which caused controversy for many years, could most likely be attributed to differences in experimental conditions (the flow rate of the water through the magnetic field, the impurities in the water, and the distribution of the magnetic field) and other subtle effects (if any) that which are easily hidden by measurement errors. Despite these controversies arguments, the increased evaporation rate of water in a magnetic field is a somewhat less disputed phenomenon [7–9]. Nakagawa *et al*. [7] reported the enhancement in the evaporation of pure water in a gradient magnetic field and proposed that the enhanced evaporation was caused by convection driven by the magnetization force. Wu *et al*. [10] reported that the evaporation of pure water was increased inside a static magnetic field (0.25, 0.36 and 0.55 T) when compared to evaporation outside the magnetic field. Furthermore, the amount of evaporation was found to be dependent on the magnitude of the magnetic field: a higher magnetic field corresponds to a greater amount of evaporation. Szczes *et al*. [11] reported enhancement of water evaporation after the water flowed through a magnetic field. Interestingly, the water seems to “remember” the process of passing through the magnetic field, a phenomenon that referred to as the “memory effect”. Szczes *et al*. also pointed out that the amount of evaporation was related to the flow rate through a magnetic field of 0.27 T.

Although a few studies have been completed on the effects of magnetic fields on the evaporation of water, there is no systematic study specifically addressing the effects of the magnetic field gradient and the magnetic field itself (including the evaporation phenomenon of water under simulated microgravity generated by a large gradient magnetic field). Furthermore, the explanations of the mechanism underlying the phenomenon remain controversial. Therefore, additional investigations of the effects of magnetic fields on the evaporation of water are still needed to further clarify the phenomena and its related mechanisms.

This paper presents an investigation of the evaporation of water in a large gradient magnetic field. Different simulated gravity positions in the magnetic field, including 0 g (simulated microgravity position, magnetic field 8.69 T), 1 g (simulated normal gravity, magnetic field 16.12 T), 1.56 g (simulated gravity 1.56 g, magnetic field 8.69 T) and 1.96 g (simulated gravity 1.96 g, magnetic field 12.64 T) were used to test the evaporation phenomenon of water for the first time. The effects of a magnetic field and a magnetic field gradient on the properties of water are discussed based on the experimental results.

## 2. Results

The evaporation of water at different specific positions both within and outside of the magnetic field was investigated. The amounts of pure water evaporated at four specific positions in the magnetic field (0 g/8.69 T, 1 g/16.12 T, 1.56 g/8.69 T, 1.96 g/12.64 T) and in the absence of the magnetic field (1 g/0 T) were compared. The resulting effects of the magnetic field, the field gradient, and their combination on the evaporation of water are presented in the following subsections.

### 2.1. Effect of Magnetic Field on the Evaporation of Water

**Figure 1:**
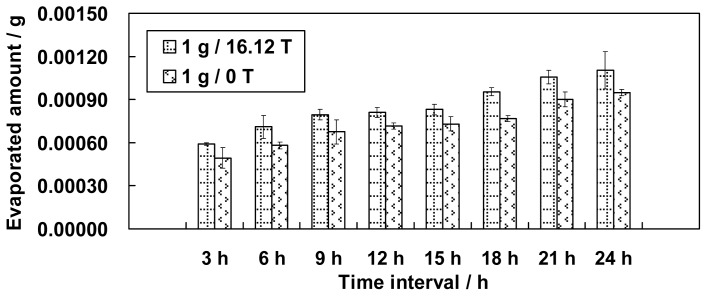
Comparison of water evaporation in a homogeneous magnetic field (at position 1 g/16.12 T) and in the absence of the magnetic field (at position 1 g/0 T) (error bars: s.e.m. (standard error of the mean), *n* = 3).

Figure 1 shows the experimental results of the measured amount of evaporated water plotted against time at positions 1 g/16.12 T and 1 g/0 T. Based on the comparison, the effect of a magnetic field on water evaporation is illustrated. The results indicated that the magnetic field enhanced the evaporation of the water under the same gravity conditions. The effect of the magnetic field on water evaporation was more significant than that of the field gradient.

### 2.2. Effect of Magnetic Field Gradient on the Evaporation of Water

The effects of the magnetic field gradient on the evaporation of water at positions 0 g/8.69 T and 1.56 g/8.69 T were compared. Because the magnetic field was the same (8.69 T), the differences in the evaporation between the two groups were caused by the differences in the simulated gravities (0 g and 1.56 g).

**Figure 2:**
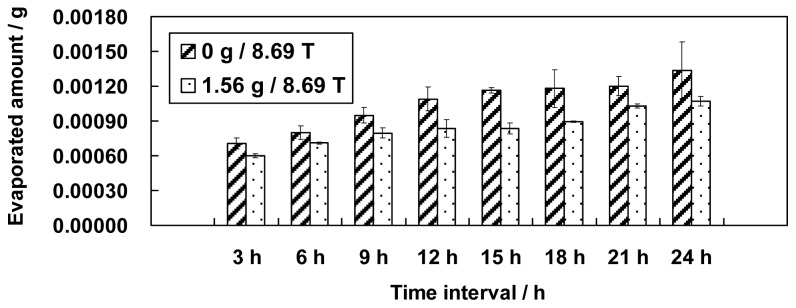
Comparison of water evaporation in simulated microgravity (at position 0 g/8.69 T) and in simulated hypergravity (at position 1.56 g/8.69 T) (error bars: s.e.m. (standard error of the mean), *n* = 3). Based on the comparison, the effect of the magnetic field gradient on water evaporation is illustrated. The results show that simulated microgravity exhibited a stronger ability to enhance the evaporation of water compared with simulated hypergravity.

Figure 2 shows that the evaporation of water could be affected by a field gradient. Different field gradients showed different effects on the amount of evaporated water. Water evaporation at simulated microgravity (0 g) conditions was increased compared with the evaporation at simulated hypergravity (1.56 g).

### 2.3. Combined Effect of Magnetic Field and Magnetic Field Gradient on the Evaporation of Water

**Figure 3:**
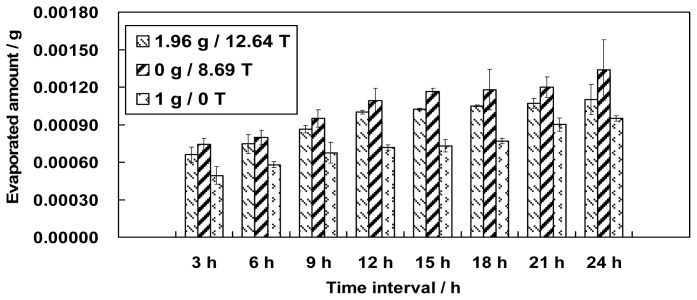
The combined effect of a magnetic field and magnetic field gradient comparing the amount evaporation at three positions (1.96 g/12.64 T, 0 g/8.69 T and 1 g/0 T) (error bars: s.e.m. (standard error of the mean), *n* = 3). The results show that simulated microgravity exhibited the highest evaporation rate and the control showed the lowest evaporation rate.

The results of the two experiments showed that both the magnetic field and the magnetic field gradient (simulated lower gravity) enhanced the evaporation of water. Further research was conducted to compare the combined effect of the magnetic field and magnetic field gradient on water evaporation to the evaporation without a magnetic field. The results of evaporation at three positions were compared: 1.96 g/12.64 T, 0 g/8.69 T, and 1 g/0 T. Figure 3 shows that the evaporation of water was facilitated by simulated microgravity (0 g/8.69 T), whereas evaporation in the absence of the magnetic field had the lowest evaporation rate.

## 3. Discussion

To understand the phenomenon of water evaporation at various magnetic field positions (0 g/8.69 T, 1 g/16.12 T, 1.56 g/8.69 T, 1.96 g/12.64 T), it is necessary to investigate the effect of the magnetic field on the physical properties of water or on the surrounding environment. A gradient magnetic field exerts greatly on water and its environment through the Lorentz force, magnetization force, and torque (if particles with inhomogeneous magnetic susceptibility exist). In addition, the gradient magnetic field has a nontrivial influence on both hydrogen bonding and van der Waals forces in water. Apparently, water evaporation in a gradient magnetic field depends on the forces mentioned above.

### 3.1. Magnetization Force Causes Different Liquid Surface Areas at Different Positions in the Magnetic Field

The magnetization force, *F_m_* acting on the materials in a gradient magnetic field, can be defined as [12],

(1) $$F_{m}=-\frac{\chi }{\mu_{0}}BB^{\prime }$$

where χ is the magnetic susceptibility of a unit volume of the material in the magnet, μ_0_ is a constant (μ_0_ = 4π × 10^−7^ H m^−1^), and *B* and *B*′ are the magnetic field and its gradient, respectively.

Water experiences repulsive forces in the magnetic field because of its intrinsic diamagnetism. Therefore, water in a container in different positions under the magnetic field exhibits different surface shapes in response to the nonhomogeneous distribution of the magnetic field in the superconducting magnet. In our current study, the center of the magnetic field holds the field gradient at *B*′ = 0 T/m, which is theoretically the same as outside the magnetic field. As a result of this magnetic field gradient, the surface shape of the water at that location will be quite similar to that of water outside the magnetic field. However, the water/air surface in a gradient magnetic field changes significantly at non-center positions.

**Figure 4:**
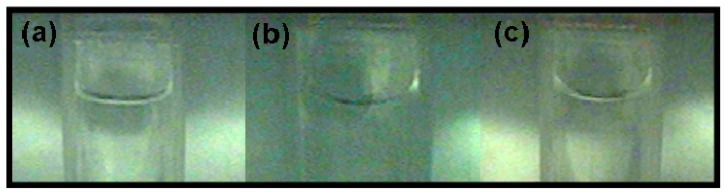
Images of the sample cells with water in the magnetic field at (**a**) position 1.96 g/12.64 T, (**b**) position 1 g/16.12 T, and (**c**) position 0 g/8.69 T.

**Figure 5:**
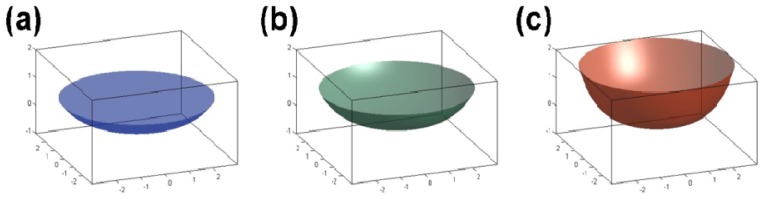
Simulated diagram of the water/air interface in the magnetic field at (**a**) position 1.96 g/12.64T, (**b**) position 1 g/16.12T, and (**c**) position 0 g/8.69T.

We captured images of the sample cell at three different positions (0 g/8.69 T, 1 g/16.12 T, and 1.96 g/12.64 T) in the magnetic field using a real-time video surveillance system. As shown in Figure 4, the surface shapes of the water/air interface vary observably at different positions. This variation depends on the interaction between the container wall and the water. Factors affecting the variation include the water purity, surface tension, *etc*., and the material of the container. The variation in surface shape also depends on the magnetization force acting on the water, which is closely related to the distribution of the magnetic field. Moreover, we measured the contact angle between the water and the container wall (the cover of the Eppendorf cube) from the image we acquired and calculated the area of the water surface. With more information about the contact angle, the shape of the water/air interface can be obtained as shown in Figure 5, and the surface area can be calculated. At the three different positions of 1.96 g/12.64 T, 1 g/16.12 T and 0 g/8.69 T, the contact angles were 12°, 17° and 23° and the corresponding surface areas were 32.08 mm^2^, 34.65 mm^2^ and 44.65 mm^2^, respectively.

The resulting relationship between position and surface area is similar to previously reported results [13]. This relationship convinces us that the magnetization force affects the amount of evaporated water, because evaporation is proportional to the evaporating surface area. However, the evaporation amounts shown in Figures 1 and 3 did not monotonically increase as expected based on the surface area.

If the amount of evaporated water is determined only by its surface area, we might have observed the largest amount of evaporation at position 0 g/8.69 T and the smallest amount of evaporation at position 1.96 g/12.64 T. The smallest amount of evaporation, however, occurred unexpectedly at position 1 g/16.12 T. The variation in the surface areas slightly conflicts with the findings in section 2 that either the increment of the magnetic field or the decrement of the gravity level can increase the amount of evaporation. We believe, therefore, that the surface area for evaporation is one but not the only factor that determines the amount of evaporation. Thus, other factors need to be explored.

### 3.2. Effect of Magnetic Field on Hydrogen Bonds and van der Waals Force

Water evaporation is a gradual process in which water molecules escape from the liquid to the surrounding environment. Because hydrogen bonding is the primary intermolecular force that keeps water molecules in the liquid phase, evaporation must involve the breaking of hydrogen bonds, but how the magnetic field influences hydrogen bonds in water is still strongly contested [5,14–18]. Several studies have shown that hydrogen bonds were strengthened or that more hydrogen bonds were formed [5,14,15], while other studies showed that hydrogen bonds were weakened [16–18].

Toledo *et al*. [6] proposed a competition mechanism according to their experimental results and theoretical calculations. They suggested that hydrogen bonds in water can be classified into two types: intra-cluster and inter-cluster hydrogen bonds. The magnetic field could break the intra-cluster hydrogen bonds and strengthen the inter-cluster hydrogen bonds. The result is the weakening of intra-cluster hydrogen bonds and the formation of smaller clusters with stronger inter-cluster hydrogen bonds.

Regardless of how hydrogen bonds in water are affected by a magnetic field, their changes (if the changes actually occur) definitely influence the evaporation of water. Strengthened hydrogen bonds will restrain water evaporation, whereas weakened hydrogen bonds will enhance water evaporation. An enhancement of evaporation from position 1 g/0 T to 1 g/16.12 T in our results demonstrated that hydrogen bonding, or its parts, may be weakened or even broken by the forces of the magnetic field. We suggest, therefore, that the weakening of hydrogen bonds in the magnetic field contributes to the amount of evaporation.

Additionally, van der Waals forces between the water molecules provide a boost for holding the molecules together. The van der Waals forces, however, are weaker than hydrogen bonds. The weakening of van der Waals forces was regarded as a reason for the enhancement of water evaporation in a magnetic field but not outside a magnetic field. Because weak van der Waals complexes can be broken inside a magnetic field through coupling between the Zeeman energy levels [19], the weakening of van der Walls forces in water can be regarded as another aspect contributing to increased water evaporation with the increment of the magnetic field.

Therefore, the weakening of hydrogen bonding and van der Waals forces between water molecules caused by a magnetic field makes it easier for water molecules to escape from the surface, and can help explain the change in the amount of evaporation between water in the absence and the presence of a magnetic field.

### 3.3. Effect of Field Gradient on the Convection near the Liquid/Gas Interface during Evaporation

Our previous results led to a potential conclusion that either the increment of the magnetic field or the decrement of the gravity level can increase the evaporation amount, and vice versa. Unexplained by the results, however, are the high amount of evaporation at the 1.96 g/12.64 T magnetic field and the low amount of evaporation at the 1 g/16.12 T magnetic field. This interesting phenomenon can be interpreted by the convection in the magnetic field that drives the evaporation [7,8]. According to the distributions of the magnetic field and its gradient, the air molecules at the locations both near and distant from the water’s surface are exerted by the varied magnetization forces. The variation of the magnetic force, Δ*F_m_*, can be defined using Equation 2:

(2) $$\Delta F_{m}=-\frac{\Delta \chi }{\mu_{0}}BB^{\prime }$$

Where Δχ = χ_air_ − χ_wet_ = 0.0088 × 10^−6^ and depends on the susceptibilities of the air composition [7]. Because different compositions in the air near the liquid/gas interface (including oxygen, nitrogen and water vapor) have distinct magnetic susceptibilities, they are exerted on by different magnetization forces even at the same position in a magnetic field. The paramagnetic volume susceptibilities of oxygen, nitrogen and water vapor are χ_O_2__ = 1.9 × 10^−6^, χ_N_2__ = −5 × 10^−9^ and χ_H_2_O_vapors__ = −6.8 × 10^−^, respectively where a negative value indicates that the gas is diamagnetic. Therefore, Δχ in Equation 2 has a large contribution from oxygen due to its larger paramagnetic susceptibility as opposed to the diamagnetic nitrogen or water vapor.

Importantly, Δ*F_m_* is positively correlated to the convection, which definitely affects the speed of evaporation [7,8]. In our superconducting magnetic field, *BB*′ had a greater value in larger field gradient positions (0 g/8.69 T and 1.96 g/12.64 T) than that in smaller field gradient positions (1 g/16.12 T and 1.56 g/8.69 T), and produced a larger Δ*F_m_*, which then accelerated the convection near the liquid/gas interface. Consequently, the evaporation in larger field gradient positions was enhanced. This situation arose from the acceleration of convection that was observed.

In conclusion, the amount of water evaporation depends on the surface area of the liquid/gas interface, the change in intensity of hydrogen bonding and van der Waals forces, and the speed of convection near the liquid/gas interface.

Therefore, the enhancement of water evaporation in a magnetic field can be explained by Equation 3:

(3) $$W_{evaporation}=f(A)+f(B)+f(B^{\prime })$$

where *W_evaporation_* is the amount of water evaporation and is determined by *f*(*A*), *f*(*B*) and *f*(*B*′). In Equation 3,*A* is the area in the water/air interface, *B* is the magnetic field, and *B*′ is the field gradient.

## 4. Experimental Section

Experiments were conducted to investigate the effects of a static magnetic field and magnetic field gradient on the evaporation of triply water, with quantitative comparisons of water evaporation at four positions inside the magnetic field and outside the magnetic field.

**Figure 6:**
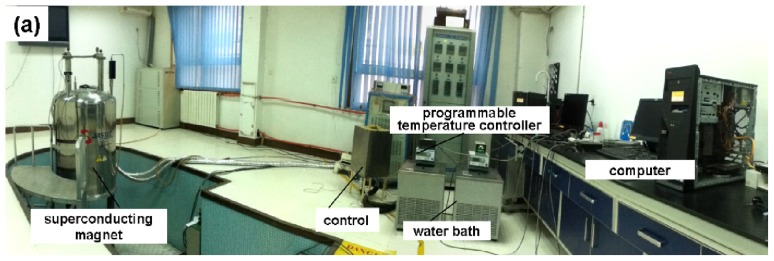

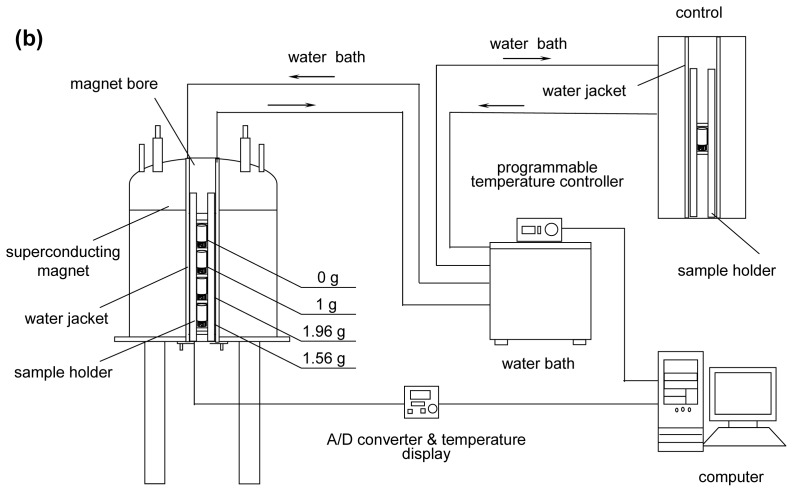
Overall configuration of the instruments with a large-gradient high-field magnet. (**a**) photograph of the system and (**b**) a schematic illustration of the system. Four special positions in the magnet bore (simulated 0 g, 1 g, 1.56 g and 1.96 g) and a control were utilized for placing the samples

As shown in the schematic in Figure 6, the system consists of four parts: a superconducting magnet, an experimental sample holder (to fix the samples at specific positions in the magnet bore and control the temperature of the samples), a control sample holder (to hold a control sample and control the temperature of the sample outside the magnet) and a temperature control system (including a water bath, a computer, an A/D converter, and water jackets).

A superconducting magnet (JASTEC 16T50MF, JASTEC, Kobe, Japan) was utilized to provide the magnetic field. The magnet is capable of generating a large gradient magnetic field ranging from −1500 T^2^/m to 1312 T^2^/m. The large range of gradient magnetic fields allows for the simulation of gravities ranging from microgravity (~0 g) to hypergravity (~2 g). Such gravity simulation is useful in investigations that depend on gravity change (e.g., studies related to space exploration) [13,20–27].

Simulation of gravities using a gradient magnetic field is a well-established method that makes full use of the magnetization force, Δ*F_m_* exerted on the object in the gradient magnetic field in Equation 1 [12].

Because the magnetization force is a body force, the simulated gravity (*i.e*., simulated acceleration) *g_sim_* in a gradient magnetic field can be expressed as follow:

(4) $$g_{sim}=g-\frac{\chi }{\mu_{0}\rho }BB^{\prime }$$

where: *g* is the gravitational acceleration and ρ is the density of the subject material.

According to Equation 4, the simulated gravity for water can be obtained at a specific position in the magnetic field, where the magnetic field and the magnetic field gradient are determined. According to the field distribution in the magnet, four specific simulated gravity positions (for pure water) [23] were utilized: 0 g (the simulated microgravity position in which the magnetic field was 8.69 T, labeled as 0 g/8.69 T, 331 mm away from the magnet bottom), 1 g (the simulated normal gravity, 1 g/16.12 T, 245 mm away from the bottom), 1.96 g (simulated hypergravity, 1.96 g/12.64 T, 170 mm away from the bottom), and 1.56 g (simulated hypergravity, 1.56 g/8.69 T, 133 mm away from the bottom).

By using the sample holder, the samples can be readily fixed at the four positions. The samples consisted of vessels of the same inner diameter (Φ 6.66 mm, with a volume of 0.5 mL) for filling with water. The samples would be placed into a larger sealed container (a clean, dry 4.6 mL glass bottle with an inner diameter Φ 11.52 mm) so that the space and the geometrical configuration for evaporated water was the same for all comparative experiments.

The temperature of all the samples was controlled in a water jacket (Figure 6b) in which the temperature-controlled water (at 293 ± 0.1 K) flowed using a programmable water bath circulator (Polyscience 9712, Polyscience, Niles, IL, USA).

**Figure 7:**
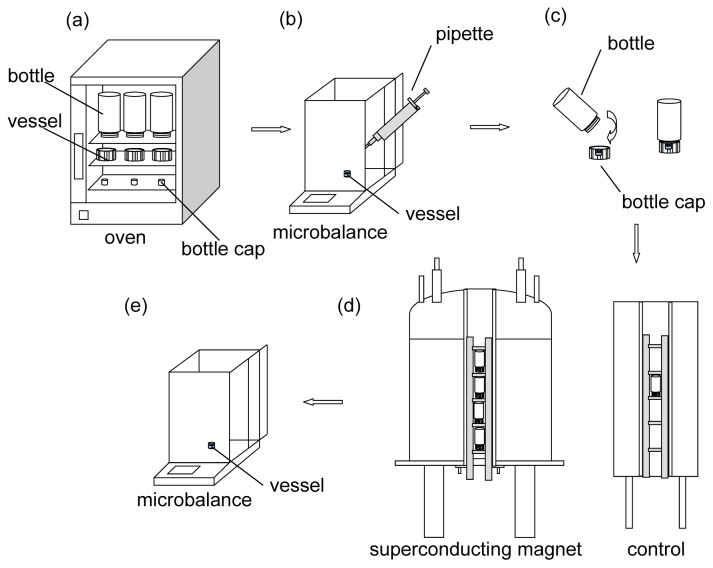
Schematic illustration of the experimental processes including (**a**) heating the containers to remove water, (**b**) filling the vessel with water and measuring the weight of the filled vessel, (**c**) placing the filled vessel into the bottle cap and sealing the bottle (the bottle is set upside down), (**d**) placing the sealed bottles into different experimental positions inside and outside the magnet, (**e**) measuring the weight of the filled vessel after evaporation and obtaining the evaporated amount by comparing it with the initial weight.

The experimental processes are shown in Figure 7. First, all containers (the bottles with their caps, and the small vessels for filling water) were cleaned and heated in an electric oven (DHC-9146A, Shanghai Jing Hong Laboratory instrument Co. Ltd., Shanghai, China) at 333 K for 2 h to remove the water vapor in the containers (Figure 7a). Second, equal volumes of water (60 μL, purified using Milli-Q Millipore System, Millipore, Germany, with a resistivity of approximately 18.2 MΩ at 298 K) were pipetted into the vessels, respectively. At the same time, the total weight of each vessel with the 60 μL of water was measured (BT125D, Sartorius, Germany, d = 0.01 mg) and recorded (Figure 7b). Third, a filled vessel was immediately placed into each cap of the bottles, and the bottles were sealed (Figure 7c). Fourth, the sealed bottles were immediately placed (with the filled vessels) at different positions in the magnetic field and allowed to evaporate (Figure 7d). Fifth, after every 3 h (*i.e*., 3, 6, 9, 12, 15, 18, 21, and 24 h after starting the experiments), the weight of each filled vessel was measured after evaporation in the magnetic field. Thus, the evaporated amount was obtained by comparing the final weight with the initial weight of the filled vessel (Figure 7e).

Note that, the experiment for each time period (*i.e*., 3, 6, 9, 12, 15, 18, 21, and 24 h) was carried out separately, meaning that each filled vessel was disposed after its second weight measurement. In other words, none of the experiments were interrupted during the evaporation to avoid any errors caused such the interruptions.

Outside the magnetic field, a control evaporation experiment (1 g/0 T) was conducted simultaneously using the conditions and protocol as described above.

Apart from the evaporation experiments, we also conducted an experiment to visualize the surface shape of the water at different positions in the magnet (positions at 0 g/8.69 T, 1 g/16.12 T, and 1.96 g/12.64 T) to provide more information about the effect of the surface area on the evaporation. The real-time observation system [20,21] consisted of a CCD controller, a CCD detector (Ultra Micro-CCD UN43H, ELMO Co., Ltd., Nagoya, Japan), an image acquisition software (Ulead Video Studio 9.0, Corel Corporation, Ottawa, Canada, 2012), a control acquisition software (Quick Macro 9.0, Brothers software, Fuzhou, China, 2012), video signal transmission lines and a signal transformation equipment. The sample image was obtained through a mirror placed beside the sample cell. Because the vessel utilized in the evaporation experiment was too small to be observed by the Micro-CCD camera, we utilized a larger sample cell (12 mm × 12 mm × 45 mm) that was composed of the same type of materials (polyethylene) as that of the vessels utilized in the evaporation experiment. Because the replacement cell was made from the same material, the contact angle between the water and the container should remain the same at the same position. A mirror was placed beside the cell at an angle of 45° against the central axis of the cell so that the Micro-CCD camera could acquire the vertical images of the cell from above the mirror.

With the contact angle information obtained using actual images at different positions, we were able to estimate the surface areas of the water in the vessels at different positions in the evaporation experiment. Because the image we acquired showed only a cross section of the water and the sample cell, further treatment was necessary to obtain the information for the surface area of the water. Fortunately, the axial symmetry of the vessels allows for the feasible measurement of the surface area of the water. The treatments we adopted are described as follows:

1. Mark a series of points along the liquid/air interface in each image by the integration of automatic image enhancement and manual marking.
2. Map the positions of all marked points in each image into a Cartesian coordinate system of which the origin is the lowest point in the image. Next, perform a polynomial curve fitting on their coordinates to get a curve corresponding to the cross section of the liquid surface.
3. Obtain the contact angle by calculating the tangent slope of the fitted curve at the contact position.
4. Calculate the surface area by integral operation. The liquid surface can be formed by rotating the fitted curve around the central axis of the vessel.

Here, we give the final practical formula for the area calculation as Equation 5:

(5) $$Area=2\pi \int_{o}^{r}x\sqrt{1+{\left(y^{\prime }\right)}^{2}}\text{d}x$$

where *y* is the polynomial of the fitted curve corresponding to the cross section of liquid surface and *r* is the radius of the sample cell. Because it is always hard to get an explicit integral, we apply the adaptive Lobatto quadrature to numerically evaluate the integral formula [28].

## 5. Conclusions

The amount of water evaporated at different positions both within and in the absence of a magnetic field were investigated. The enhancement of water evaporation in a magnetic field was verified. Furthermore, it was found that the amount of evaporation was related to the position in the magnetic field. Water at the simulated microgravity position (0 g) exhibited the largest amount of evaporation, water at simulated hypergravity (1.96 g) also exhibited enhanced evaporation, whereas water in the absence of the magnetic field exhibited the least amount of evaporation. The phenomena were related to the different areas of the water/air interface and the different magnetization forces and the field gradients at different positions in the magnetic field. The effects of the magnetic field on hydrogen bonds and van der Waals forces were also discussed, but have not yet been verified due to contradictory reports.
